# Development and Characterization of a Hydrogel Incorporating Protamine–Hyaluronic Acid Nanoparticles Co-Loaded with Disulfiram and Coumarin for Diabetic Wound Healing

**DOI:** 10.3390/pharmaceutics18091180

**Published:** 2026-09-18

**Authors:** Zainab Lafi, Mohammad I. A. Ahmad, Shreen Deeb Nusair, Razan Madi, Alaa Al-Sanabrah, Somaya Ahmad, Qout Qtashat, Maisrh Ali Alrubaye, Sara Yousef Asha

**Affiliations:** 1 Pharmacological and Diagnostic Research Center, Faculty of Pharmacy, Al-Ahliyya Amman University, Amman 19328, Jordan; m.malkawy@ammanu.edu.jo (M.I.A.A.); r.madi@ammanu.edu.jo (R.M.); a.sanabrah@ammanu.edu.jo (A.A.-S.); s.alhaq@ammanu.edu.jo (S.A.); q.qtashat@ammanu.edu.jo (Q.Q.); maisrhalrubaye@gmail.com (M.A.A.); 2 Department of Pharmaceutics and Pharmaceutical Technology, Al-Ahliyya Amman University, Amman 19328, Jordan; 3 Faculty of Allied Medical Sciences, Al-Ahliyya Amman University, Amman 19328, Jordan; 4 Department of Clinical Pharmacy, Faculty of Pharmacy, Jordan University of Science and Technology, P.O. Box 3030, Irbid 22110, Jordan; sdnusair@just.edu.jo; 5 School of Medicine, Jordan University, Amman 19328, Jordan; sarah.asha20@gmail.com

**Keywords:** protamine, hyaluronic acid, nanoparticles, disulfiram, coumarin, hydrogel, diabetic wound healing, drug delivery

## Abstract

**Background:** Diabetes-associated wound healing is frequently impaired due to persistent hyperglycemia, which causes vascular dysfunction and neuropathy, thereby delaying tissue repair. **Objectives:** In this study, hyaluronic acid and protamine nanoparticles (HA-PRO-NPs) were developed and optimized to encapsulate disulfiram (DSF) and coumarin (COM) for potential application in diabetic wound healing. **Methods:** The nanoparticles were prepared using a simple ionic interaction method and characterized for their particle size, zeta potential, morphology and encapsulation efficiency. Then, HA-PRO-NPs were incorporated into a hydrogel that contains carboxymethyl cellulose (CMC) and propylene glycol (PG). The hydrogel formulations were evaluated for swelling behavior, spreadability and DSF and COM release kinetics. **Results: **The prepared formulations were safe against human dermal fibroblasts (HDFs), with cell viability remaining above 70% over the concentration ranges tested. In the scratch assay, HA-PROT-DSF-COM-NPs improved HDF migration, achieving approximately 81% wound closure. An in vivo study was conducted using streptozotocin-induced diabetic mice. Topical application of Gel-HA-PROT-DSF-COM-NPs promoted wound closure. The study monitored wound contraction on Days 0, 3, 7, and 12. By Day 12, topical administration of the dual-cargo system (Gel-HA-PROT-DSF-COM NPs, n = 6) achieved 78.2 ± 4.1% wound closure. This was significantly higher than the single-drug formulation (Gel-HA-PROT-DSF-NPs: 59.5 ± 4.8%) and the untreated Control (Diabetic) group (55.4 ± 5.2%, *p* < 0.01), while the non-diabetic Control group (non diabetic) reached 97.6 ± 3.5% closure. Semi-quantitative histopathological evaluation showed that the Gel-HA-PROT-DSF-COM NPs achieved a healing score of 4/5, characterized by optimized epidermal regeneration with preserved adnexal structures and moderate dermal remodeling. **Conclusions:** In conclusion, Gel-HA-PROT-DSF-COM-NPs demonstrated promising potential as a topical formulation for improving wound healing under diabetic conditions.

## 1. Introduction

Wound healing is a multi-stage physiological phenomenon involving several overlapping phases including hemostasis, inflammation, proliferation, and tissue remodeling [1,2]. Delayed wound healing presents many clinical problems especially in diabetic patients [3]. The healing process is markedly disrupted by persistent hyperglycemia, impaired vascular function, neuropathy, increased oxidative stress, and chronic inflammation [4]. Together, these factors slow tissue repair and increase the risk of wound infection. As a result, diabetic wounds often develop into chronic ulcers, which remain a major cause of morbidity and lower-limb amputation [5]. Consequently, there is a need to develop an advanced drug delivery system capable of releasing components for a prolonged duration in individuals with diabetes mellitus. Although considerable progress has been made in developing advanced therapeutic approaches, the molecular mechanisms underlying diabetic wound healing are still not fully understood [6]. Drug delivery nanoparticles (NPs) can provide sustained and localized therapeutic effects to promote tissue regeneration, reduce infection, and accelerate wound healing [7,8].

Hyaluronic acid (HA) plays a vital role in the wound-healing process due to its excellent biocompatibility, biodegradability, and ability to retain large amounts of water [7,9]. As a naturally occurring component of the extracellular matrix, HA helps maintain a moist wound environment, which is essential for efficient tissue repair and regeneration. It supports key healing events by promoting cell migration, proliferation, and angiogenesis, while also facilitating communication between cells involved in tissue reconstruction [9]. Hyaluronic acid can modulate inflammatory responses, helping to reduce excessive inflammation that may delay healing. Its unique physicochemical properties have made it a widely used biomaterial in wound dressings, hydrogels, and drug-delivery systems aimed at accelerating wound closure and improving overall healing outcomes, particularly in chronic and hard-to-heal wounds [1,10,11].

Among naturally derived biomaterials, protamine has emerged as a promising candidate for wound-healing applications because of its excellent biocompatibility and inherent antimicrobial activity [12]. Protamine is a positively charged peptide composed predominantly of arginine residues (approximately 65–70%), with smaller amounts of proline, serine, valine, glycine, and alanine. Depending on its source, its molecular weight typically ranges from 4 to 5 kDa. The high density of positively charged arginine residues enables protamine to interact readily with negatively charged biomolecules, a property that facilitates nanoparticle formation, efficient drug encapsulation, and localized drug delivery. These characteristics have made protamine an attractive carrier for nanoparticle-based drug delivery systems and wound-healing formulations [13]. In wound healing applications, protamine may help reduce microbial contamination, promote tissue repair, and improve the localized delivery of therapeutic agents. Owing to these multifunctional properties, protamine has emerged as a promising biomaterial for the development of advanced wound dressings and regenerative therapies [14].

Disulfiram has demonstrated anti-inflammatory and antimicrobial activities, whereas coumarin has been reported to promote cell proliferation and tissue repair. Advances in nanotechnology offer effective strategies for enhancing drug delivery by enabling controlled release, improving tissue penetration, and facilitating localized delivery to the wound site [15,16,17]. Sun et al. 2024 developed a hydrogel from sodium alginate that included DSF for wound healing in diabetic wounds. They revealed that the formulation decreased inflammation by reducing the formation of neutrophil extracellular traps (NETs), suppressing the Caspase-1/GSDMD inflammatory pathway, and promoting M2 macrophage polarization. It also enhanced angiogenesis and re-epithelialization, accelerating wound closure [18]. On the other hand, ref. [19] Dutra et al. (2024) investigated the wound-healing potential of two coumarin derivatives, formulated as β-cyclodextrin (β-CD) inclusion complexes. They demonstrated that complex formation enhanced the solubility and stability of coumarins. In vivo studies in mice revealed that the coumarin/β-CD accelerated re-epithelialization and collagen deposition. They reduced granulation tissue and mast cell density while improving collagen remodeling. This highlighted its potential as a promising coumarin-based approach for enhancing wound repair.

The present study aimed to develop a novel nanoparticle (NP) composite system incorporating disulfiram DSF and COM within hyaluronic acid and protamine-based nanoparticles. The nanoparticles were prepared using an ionic interaction method and subsequently embedded in a biocompatible hydrogel composed of carboxymethyl cellulose (CMC) and propylene glycol (PG) to enhance topical delivery and wound retention.

## 2. Materials and Methods

### 2.1. Materials

Hyaluronic acid sodium salt (30–50 kDa) was purchased from Genochem World (Valencia, Spain). Protamine sulfate (from salmon sperm) was obtained from TCI Chemicals (Tokyo, Japan). Streptozotocin (STZ; Cat. No. MS07936) was supplied by Biosynth (Staad, Switzerland). Disulfiram (DSF), Coumarin, sodium citrate tribasic dihydrate, methanol, Carboxymethyl cellulose sodium salt (CMC, moderate viscosity) and ethanol (analytical grade) were purchased from Sigma-Aldrich (St. Louis, MO, USA). All other chemicals and reagents were of analytical grade and used as received without further purification. Deionized water was used throughout all experiments.

### 2.2. Methods

#### 2.2.1. High-Performance Liquid Chromatography (HPLC) Analysis of Disulfiram and Coumarin

The simultaneous quantification of DSF and COM was performed using an HPLC system (Shimadzu Corporation, Kyoto, Japan) equipped with a UV detector. Chromatographic separation was achieved on a reversed-phase C18 column (Shim-pack GI, Kyoto, Japan) (100 Å, 5 μm, 4.6 × 150 mm). The mobile phase consisted of methanol and water (80:20, *v*/*v*), with the aqueous phase adjusted to pH 3.0 [20]. Prior to analysis, the mobile phase was filtered through a 0.45 μm membrane filter (Visking dialysis tubing with a molecular weight cut-off of 12,000–14,000 Da (Medicell Membranes Ltd., London, UK) and degassed. The HPLC system was operated at a flow rate of 1 mL/min with an injection volume of 10 μL. The total run time was 6 min, and detection was carried out at 275 nm. Mixed stock solutions containing both DSF and COM were prepared in methanol at a concentration of 1 mg/mL for each analyte. The mixed stock solution was serially diluted with methanol to obtain calibration standards containing both compounds at concentrations of 0.002–1 mg/mL. Each calibration standard was analyzed in triplicate, and calibration curves were constructed by plotting the peak area of each analyte against its corresponding concentration. Linear regression equations were generated and used for the simultaneous quantification of DSF and COM in NP formulations and hydrogel formulations. The adapted HPLC method was successfully validated using our established institutional infrastructure and identical instrument configurations, demonstrating high reliability and reproducibility for the concurrent quantification of both drugs. The method exhibited excellent specificity, with no interfering peaks from the blank HA-PROT nano-matrix or the solvent front at the retention times of COM (3.138 min) and DSF (4.173 min). Strong linearity was confirmed over a wide working range of 1–1000 µg/mL for both analytes, yielding linear regression equations of (y = 30,936x\R^2^ = 0.9999) for COM and (y = 16,288x\R^2^ = 1.000) for DSF. Method accuracy and precision were highly satisfactory, yielding recovery rates between 98.5% and 101.2% alongside intra- and inter-day precision values consistently below 2.0% RSD. The limits of detection (LOD) and quantification (LOQ) were determined to be 0.45 µg/mL and 1.35 µg/mL for DSF, and 0.25 µg/mL and 0.75 µg/mL for COM, respectively. Furthermore, robust system suitability was maintained (replicate injection peak area RSD < 1.0%, tailing factors < 1.5, and theoretical plate counts > 3000), and both analytes remained highly stable (>98% recovery) under autosampler (24 h) and storage (4 °C for 7 days) conditions.

#### 2.2.2. Preparation of Hyaluronic Acid–Protamine Nanoparticles

DSF- and COM-loaded HA-PROT-NPs were prepared using the ionic complexation method with sodium citrate as a stabilizing agent. Hyaluronic acid sodium salt was dissolved in deionized water to obtain a concentration of 10 mg/mL under magnetic stirring at 700 rpm at room temperature. Sodium citrate was then added to the HA solution at a concentration of 1 mg/mL and stirred until completely dissolved. Separately, DSF (1 mg) and COM (0.5 mg) were co-dissolved in 1 mL of ethanol. This organic drug phase was added dropwise into 1 mL of the HA/sodium citrate solution (containing 10 mg of HA) under continuous stirring to form a HA/drug dispersion. Protamine sulfate was separately dissolved in deionized water at a concentration of 5 mg/mL. Finally, 1 mL of the protamine solution (containing 5 mg of PROT) was added dropwise to the HA/drug dispersion to maintain a strict HA to PROT mass ratio of 2:1, bringing the total formulation volume to exactly 3 mL. The electrostatic interaction between the negatively charged carboxyl groups of HA and the positively charged guanidinium groups of protamine promoted the spontaneous formation of nanoparticles. The resulting 3 mL suspension was stirred for an additional 30 min at room temperature to ensure complete nanoparticle formation and efficient drug encapsulation [21].

#### 2.2.3. Determination of Encapsulation Efficiency (EE%) and Drug Loading (DL%)

To separate unencapsulated drugs from the nanoparticles, equilibrium dialysis was performed. Briefly, 3.0 mL of the freshly prepared HA-PROT-NP suspension (containing a nominal feeding dose of 1.0 mg DSF and 0.5 mg COM) was placed inside a dialysis membrane pouch (MWCO 12–14 kDa, (Medicell Membranes Ltd., London, UK). The pouch was immersed in an appropriate release medium under continuous stirring at 37 °C to allow free, unencapsulated drugs to equilibrate into the dialysate. The concentration of free drugs in the dialysate was quantified using a validated HPLC method. To obtain the final hydrogel quantities (4.0 mg DSF and 2.0 mg COM), a 4-fold volume scaling factor was utilized during hydrogel incorporation. Following dialysis to remove unencapsulated drugs, 200 μL of the nanoparticle suspension was mixed with 800 μL of acetonitrile to disrupt the nanoparticles completely and extract the encapsulated drug. The mixture was vortexed thoroughly and sonicated, when necessary, to ensure complete extraction. The resulting solution was filtered through a 0.22 μm syringe filter, Sigma-Aldrich are manufactured under their premier filtration brand, Millipore (Burlington, MA, USA), before analysis by a validated HPLC method. The amount of encapsulated DSF was quantified using the calibration curve, and the encapsulation efficiency and drug loading were calculated according to the following equations:

$$EE\%=\frac{Amount\;of\;encapsulated\;drug\;(mg)}{Total\;amount\;of\;drug\;added\;(mg)}\;\times \;100\%$$

$$DL\%=\frac{Amount\;of\;encapsulated\;drug\;(mg)}{Total\;wiehgt\;of\;nanoparticles\;(mg)}\times 100\%$$

#### 2.2.4. Particle Size, Polydispersity Index, and Zeta Potential

The average particle size, polydispersity index (PDI), and zeta potential were measured by dynamic light scattering (DLS) at 25 °C. Before measurement, 50 μL of nanoparticle suspension was diluted with 950 μL of deionized water. All measurements were performed in triplicate, and the results are presented as mean ± standard deviation.

#### 2.2.5. Stability Study

The physical stability of the NPs was evaluated over one month of storage at 4 ± 2 °C. At predetermined time points, particle size, PDI, and zeta potential were measured. Before each analysis, the suspensions were gently vortexed to redisperse any loosely associated aggregates. Measurements were conducted using our laboratory’s Malvern Zetasizer Nano system (headquartered in the Worcestershire, UK) equipped with a 4.0 mW Helium-Neon (He-Ne) gas laser source (λ = 633 nm). The sample analysis was executed under standard SOP parameters (mansettings. nano), utilizing a clear disposable zeta cell maintained at a controlled temperature of 25.0 °C. The automated system optimized data acquisition at an attenuator setting of 11 to ensure maximal light transmission through the aqueous medium (dispersant refractive index: 1.330; viscosity: 0.8872 cP).

#### 2.2.6. Morphological Characterization

The morphology of the nanoparticles was examined using transmission electron microscopy (TEM) (SPI supplies, West Chester, PA, USA). A drop of diluted nanoparticle suspension was deposited onto a carbon-coated copper grid and allowed to adsorb for 1–2 min. Excess liquid was carefully removed with filter paper, followed by negative staining with 2% phosphotungstic acid when applicable. After air drying, the samples were observed under TEM at an appropriate accelerating voltage, and approximately 200 particles were analyzed.

#### 2.2.7. Fourier Transform Infrared Spectroscopy (FTIR)

The nanoparticle suspension was freeze-dried for 24 h using 5% (*w*/*v*) sucrose as a cryoprotectant. FTIR spectra of HA, PROT, DSF, and COM physical mixture and HA-PROT-DSF-COM-NPs were recorded over the range of 4000–400 cm^−1^ using potassim bromide (KBr, FT-IR grade) was purchased fromSigma-Aldrich (St. Louis, MO, USA) KBr pellet method, to evaluate potential interactions among the formulation components.

#### 2.2.8. In Vitro Drug Release from Nanoparticles

The in vitro release profiles of DSF and COM were evaluated using the dialysis bag diffusion method with a dialysis membrane (12–14 kDa molecular weight cut-off). Three distinct groups were evaluated: an unencapsulated free powder control dissolved in 1.0 mL of an ethanol/PBS buffer mixture, a 1.0 mL nanoparticle suspension, and a 10 mg hydrogel sample uniformly dispersed in 1.0 mL of buffer, with each system containing a precise mass equivalent to 1.0 mg of DSF and 0.5 mg of COM. Each formulation bag was immersed in 20 mL of PBS (pH 7.4) containing 0.5% (*v*/*v*) Tween 80 to maintain strict sink conditions, ensuring maximum potential concentrations remained safely below the saturation solubility boundaries of both hydrophobic payloads. The release medium was maintained at 37 ± 0.5 °C with continuous shaking at 100 rpm. At fixed intervals over a 60 h timeline, 0.5 mL aliquots of the release medium were withdrawn and immediately replaced with an equal volume of fresh, pre-warmed medium. To prevent quantitative errors from cumulative volume depletion, the concentrations of liberated DSF and COM were mathematically adjusted using a standard sampling volume correction and quantified using a validated HPLC method. Furthermore, the chemical stability of both drugs was verified in the medium at 37 °C, and the cumulative release data for each drug were interpreted separately by fitting into the mathematical Higuchi kinetics model to evaluate matrix-controlled Fickian diffusion. The concentrations of released DSF and COM were determined by HPLC. The cumulative drug release was calculated using the following equation:

(1) $$Release\%=\frac{Amount\;of\;drug\;released\;(mg)}{Total\;amount\;of\;encapsulated\;drug\;(mg)}\;\times \;100\%$$

### 2.3. Preparation of Hydrogel Loaded Nanoparticles

The nanoparticle-loaded hydrogel was prepared on a weight-by-weight (% *w*/*w*) basis to yield a total final formulation mass of 5.0 g. Briefly, carboxymethyl cellulose (CMC, 100 mg; representing 2% *w*/*w* of the final gel) was uniformly dispersed into 5.0 mL of the previously prepared aqueous HA–PROT-NP suspension under continuous magnetic stirring at 40–50 °C until complete hydration was achieved. This suspension served as the vehicle for either the single-drug or combination nanoparticle variants, with each 5.0 g hydrogel batch incorporating a payload of 4.0 mg DSF and 2.0 mg COM, yielding final matrix concentrations of 0.80 mg/g (DSF), 0.40 mg/g (COM), and 3.0 mg/g (total HA-PROT nano-carrier mass). Propylene glycol (PG; 242 µL, corresponding to 4% *w*/*w*) was then incorporated dropwise under continuous stirring to enhance structural consistency and moisture retention. The resulting mixture was cooled to room temperature and allowed to equilibrate for several hours to ensure complete gel network assembly. The pH was measured and it was ≈ (6.47 ± 0.24, n = 3) to ensure compatibility with the wound environment, and all procedures were carried out under aseptic conditions. The ionic strength (I) of the hydrogel formulation was calculated using the equation:

(2) $$I=\frac{1}{2}\sum c_{i}z_{i}^{2},$$

where $c_{i}$ is the molar concentrations and ($z_{i}$) is the valencies of sodium citrate and protamine sulfate. Based on the used quantities, the net ionic strength was estimated to be around 12–15 mM, preserving an environment suitable for electrostatic complexation [22].

#### 2.3.1. Hydrogel Swelling Study

The swelling behavior of the hydrogel loaded with NPs was evaluated using the gravimetric method. The hydrogel loaded with NPs was dried in an oven at 70 °C and weighed. A known weight (W_0_) of uniform-dimension dry hydrogel was immersed in distilled water at 25 ± 0.5 °C. The structural integrity of the hydrogel composites was evaluated at predetermined time intervals (t = 0.25, 0.5, 1, 4, 6, 8, and 12 h). At each time interval, the samples were removed, gently blotted with filter paper to remove excess surface liquid, and weighed immediately (W_t_). The hydrogels were then returned to fresh distilled water until equilibrium swelling was achieved.

The swelling ratio was calculated as follows:

$$Swelling\;Ratio=\frac{The\;weight\;of\;at\;any\;time\;(Wt)-The\;wieght\;of\;drired\;gel\;(W_{0})}{The\;wieght\;of\;drired\;gel\;(W_{0})}\;\times \;100\%$$

#### 2.3.2. The Spreadability of the Optimized Nanoparticle-Loaded CMC-PG Hydrogel

The spreadability of the optimized hydrogel loaded with NPs was determined using a plate apparatus to evaluate its topical application performance. A standardized mass of hydrogel 0.5 g was placed precisely at the center of a pre-weighed glass plate. A second identical glass plate was gently lowered vertically onto the hydrogel mass to sandwich the sample between the two surfaces and was allowed to stabilize for 1 min. Subsequently, a weight of 50 g was carefully positioned at the center of the upper glass plate. Then it was left undisturbed for 5 min to allow the hydrogel to spread outward and reach equilibrium. The final spread diameter of the compressed hydrogel circle was measured in millimeters. All measurements were conducted in triplicate (n = 3) at room temperature.

The spreadability index (SI) was calculated using the following equation:

$$SI=\frac{the\;total\;weight\;tied\;to\;the\;upper\;plate\;\times \;final\;spread\;area\;or\;diameter\;}{The\;time\;taken\;in\;seconds}\;$$

#### 2.3.3. In Vitro Drug Release from Hydrogel-Loaded NPs

The in vitro release profiles of DSF and COM from the hydrogel matrix were evaluated using the dialysis bag method. An exact mass of 10 mg of the hydrogel formulation—containing 1.0 mg of DSF and 0.5 mg of COM—was dispersed in 1.0 mL of buffer within a dialysis membrane (12–14 kDa molecular weight cut-off). The sealed membrane bag was fully immersed in 20 mL of PBS (pH 7.4) containing 0.5% (*v*/*v*) Tween 80 as the external dissolution medium. The system was maintained at 37 ± 0.5 °C with continuous shaking at 100 rpm. This setup verified strict sink conditions, keeping maximum theoretical concentrations well below the saturation solubility boundaries of both hydrophobic payloads within the system. At predetermined time intervals over a 60 h period, 0.5 mL aliquots of the external release medium were withdrawn and immediately replaced with an equal volume of fresh, prewarmed medium. To prevent quantitative errors from cumulative volume depletion, the measured drug concentrations at each time point were mathematically adjusted using a standard sampling volume correction. The concentrations of liberated DSF and COM were quantified using the validated HPLC method, and the chemical stability of both components in the medium was successfully verified at 37 °C. To determine the underlying mass transport mechanisms, the experimental cumulative release data for each therapeutic agent were interpreted separately by fitting them into the mathematical Higuchi kinetics model to evaluate matrix-controlled Fickian diffusion.

All experiments were performed in triplicate, and the cumulative percentage of drug released was calculated using the following equation:

$$Release\%=\frac{Amount\;of\;drug\;released\;(mg)}{Total\;drug\;encapsulated\;(mg)}\;\times \;100\%$$

### 2.4. In Vitro Biological Assessment

#### 2.4.1. Cell Cytotoxicity Assay (MTT Assay)

The cytotoxicity of the developed formulations was evaluated using the MTT assay on human dermal fibroblast (HDF: ATCC PCS-201-010) cells. Cells were cultured in Dulbecco’s Modified Eagle Medium (DMEM) supplemented with 10% fetal bovine serum (FBS) and 1% penicillin–streptomycin and maintained at 37 ± 0.5 °C in a humidified atmosphere containing 5% CO_2_. For the assay, HDF cells were seeded into 96-well plates at a density of approximately 1 × 10^4^ cells per well and allowed to attach overnight. The culture medium was then replaced with fresh medium containing different concentrations 0.7–50 µM of the DSF and COM NPs, free drugs, HA-PROT-NPs and cells treated with culture medium as the control group. After 72 h of incubation, 20 µL of MTT solution 5 mg/mL in PBS was added to each well and incubated for an additional 3 h at 37 °C. The formed formazan crystals were dissolved using 100–150 µL of DMSO, and the absorbance was measured at 570 nm using aBioTek microplate ELISA reader (BioTek Instruments, Winooski, VT, USA).

#### 2.4.2. In Vitro Wound Healing and Scratch Assay

The in vitro wound healing potential of the developed formulations was evaluated using a scratch assay on HDF cells. Cells were cultured in Dulbecco’s Modified Eagle Medium (DMEM) supplemented with 10% fetal bovine serum (FBS) and 1% penicillin–streptomycin and maintained at 37 ± 0.5 °C in a humidified atmosphere containing 5% CO_2_.

HDF cells were seeded into 12-well plates at a density of 1 × 10^6^ and allowed to grow until reaching approximately 90–100% confluence. A linear scratch was created in the cell monolayer using a sterile 200 µL pipette tip. Detached cells and debris were removed by gently washing with PBS. The wells were then treated with HA-PROT-NPs, HA-PROT-DSF-NPs, HA-PROT-COM-NPs and HA-PROT-DSF-COM-NPs at 1.5 and 3 µM concentrations. Images of the scratch area were captured immediately after scratching (0 h) and at selected time intervals of 12, 24, 48, 72 h using an inverted phase-contrast microscope (Model Name, e.g., Eclipse Ts2; Nikon Corporation, Tokyo, Japan). The migration of cells into the scratched area was analyzed using Image J analysis software (version 1.x, National Institutes of Health, Bethesda, MD, USA; nih.gov).

The percentage of wound closure was calculated using the following equation:

$$\mathrm{Wound}\;\mathrm{Closure}\;\left(\%\right)=\frac{The\;initial\;wound\;area\;at\;0\;time-wound\;area\;at\;time}{The\;initial\;wound\;area\;at\;0\;time}\times 100\%$$

### 2.5. In Vivo Wound Healing Study in Streptozotocin (STZ)-Induced Diabetic Mouse

#### 2.5.1. Animal Ethics and Housing

All animal experiments were conducted in accordance with institutional ethical guidelines and approved protocols for the care and use of laboratory animals and were granted approval no. (AUP: AUU/2/10/2025-2026). Healthy adult male mice, BALB/c (Swiss albino: Rodent, Mus musculus), 8–10 weeks old, weighing 20–25 g, were housed under standard laboratory conditions (25 ± 2 °C, 12 h light/dark cycle) with free access to food and water. Sample size justification was determined by an a priori power analysis to achieve a statistical power of 0.80 at an alpha level of 0.05, establishing a fixed size of 6 mice per group ((n = 6) animals per group; total (N = 42) across 7 experimental cohorts). Animal allocation across groups was automated via a computer-generated simple randomization sequence. Regarding experimental groups, the study included control groups (non-diabetic and Diabetic), vehicle control (Hydrogel-HA-PROT-NPs), and MEBO as a positive control. Treatment groups included Hydrogel-HA-PROT-DSF-NPs, Hydrogel-HA-PROT-COM-NPs, and Hydrogel-HA-PROT-DSF-COM-NPs. Each group contained 6 mice to ensure statistical relevance.

#### 2.5.2. Development of a Streptozotocin-Induced Mouse Model

Experimental diabetes was induced by a single intraperitoneal injection of streptozotocin (STZ) freshly dissolved in cold citrate buffer (0.1 M, pH 4.5) at a target dose of 55 mg/kg body weight after overnight fasting. To prevent acute, sudden hypoglycemia-induced mortality, mice were provided with a 5% glucose solution for 24 h post-injection. Blood glucose levels were measured after 72 h using a calibrated tail-vein glucometer (Accu-Chek glucometer (Roche Diagnostics, Mannheim, Germany). Mice with fasting blood glucose levels ≥250 mg/dL were considered diabetic and successfully included in the study. Animals failing to meet this baseline glycemic threshold were excluded from the protocol and humanely euthanized via carbon dioxide inhalation. Following confirmation of diabetes, animals were allowed a strict 14-day stabilization interval before initiating the surgical wound protocols [23].

#### 2.5.3. Excisional Wound Model

Diabetic mice were anesthetized using an appropriate anesthetic agent mixture of xylazine (10 mg/kg) and ketamine (100 mg/kg) administered via intraperitoneal injection. To ensure rigorous pain control and postoperative care, analgesia was provided via subcutaneous injections of buprenorphine (0.05 mg/kg) administered every 12 h for the first 48 h post-surgery. The dorsal area was shaved and disinfected with 70% ethanol. A full-thickness excisional wound of 6–8 mm in diameter was created on the dorsal region using a sterile biopsy punch (disposable 4-mm, Miltex biopsy punch (Integra LifeSciences, Princeton, NJ, USA) under aseptic conditions. Following recovery from anesthesia, mice were housed individually in sterilized cages to protect the wound bed, and health parameters were logged daily using a standardized humane endpoint system. Threshold criteria requiring early euthanasia included a body weight loss > 20% or unmanageable systemic distress; no animal reached these humane boundaries or suffered premature unexpected mortality, leaving all animals fully accounted for at study completion [3].

#### 2.5.4. Treatment Application:

On day 1, the wounds were created, photographed, and their initial dimensions were measured to establish a baseline for subsequent assessments over 12 days. The respective treatments (10 mg of hydrogel twice daily) were then immediately applied to each group as part of the experimental protocol. This application frequency of 10 mg of hydrogel every 12 h corresponds to a total mass of 20 mg of hydrogel per day. Given the fixed concentrations of the active payloads within our formulation matrix, this twice-daily application represents an absolute local therapeutic payload of exactly 16.0 µg/day of DSF and 8.0 µg/day of COM. At different points (day 3, day 7, and day 12), wound measurements were taken, and the respective treatments were applied to each group. Wound photographs were also taken at each interval to visually track the healing progress. Following the final physical analysis on Day 12, all remaining animals were humanely euthanized via carbon dioxide asphyxiation followed by cervical dislocation, and the wound bed tissues were promptly excised for histology. To eliminate analytical bias, subsequent tissue processing, H&E staining, and semi-quantitative slide evaluation were executed in a fully blinded manner by an independent veterinary pathologist who was unaware of the group designations [24].

#### 2.5.5. Data Collection and Analysis and Wound Closure Calculation

Representative images from each group at different time points were included to provide a comprehensive view of the healing process. The wound area was measured using Image J software. The percentage of wound closure was calculated at each time point using the following equation:

$$\mathrm{Wound}\;\mathrm{Closure}\;\left(\%\right)=\frac{The\;initial\;wound\;area\;at\;day\;1-wound\;area\;at\;day\;12\;}{The\;initial\;wound\;area\;at\;day\;1}\times 100$$

### 2.6. Histopathological Evaluation Using Hematoxylin and Eosin Staining (E & H)

At the end of the wound healing study, histopathological examination was carried out to evaluate tissue regeneration and the quality of wound repair. On day twelve, half of the animals from each group were euthanized by an overdose of isoflurane. Full-thickness skin samples were collected from the wound area, including a small margin of the surrounding healthy tissue, and immediately fixed in 5% formaldehyde. The collected specimens were processed using standard histological procedures. Briefly, the tissues were dehydrated through a graded series of ethanol, cleared with xylene, and embedded in paraffin wax. The paraffin-embedded tissues were then sectioned at a thickness of 5 μm using a rotary microtome (Model Name, e.g., Leica RM2235; Leica Biosystems, Nussloch, Germany), and the sections were mounted on glass slides.

Then, the tissue was stained with H&E and was examined using a light microscope (Model Name, e.g., Leica DM2500; Leica Biosystems, Nussloch, Germany). Hematoxylin stained the cell nuclei blue-purple, whereas eosin stained the cytoplasm and extracellular matrix pink, allowing assessment of the cellular and structural features of the healing tissue.

The extent of wound healing was assessed according to inflammatory cell infiltration, granulation tissue formation, collagen deposition and organization, neovascularization, and re-epithelialization. The histological features were compared between the experimental groups to evaluate the effects of the formulations on tissue regeneration and wound repair.

### 2.7. Statistical Analysis

The raw data were initially assembled using Microsoft Excel. Statistical analyses and graphical presentations were performed using GraphPad Prism version 8 (GraphPad Software, San Diego, CA, USA). Data are presented as mean ± standard deviation (SD) from a sample size of (n = 6) animals per group. Because wound contraction measurements were tracked sequentially in the same animals over time, data dependencies within individual subjects were accounted for using a Two-Way Repeated Measures Analysis of Variance (ANOVA). In this model, ‘treatment cohort’ served as the between-subject independent factor, and ‘evaluation timeline (Days 0, 3, 7, and 12)’ served as the within-subject repeated parameter. Multi-group post hoc adjustments were executed using Tukey’s post hoc multiple comparison test to extract pairwise significance levels. A (*p*-value < 0.05) was considered statistically significant.

## 3. Results and Discussion

### 3.1. Development of HPLC Method for the Quantification of DSF &COM

**Figure 1:**
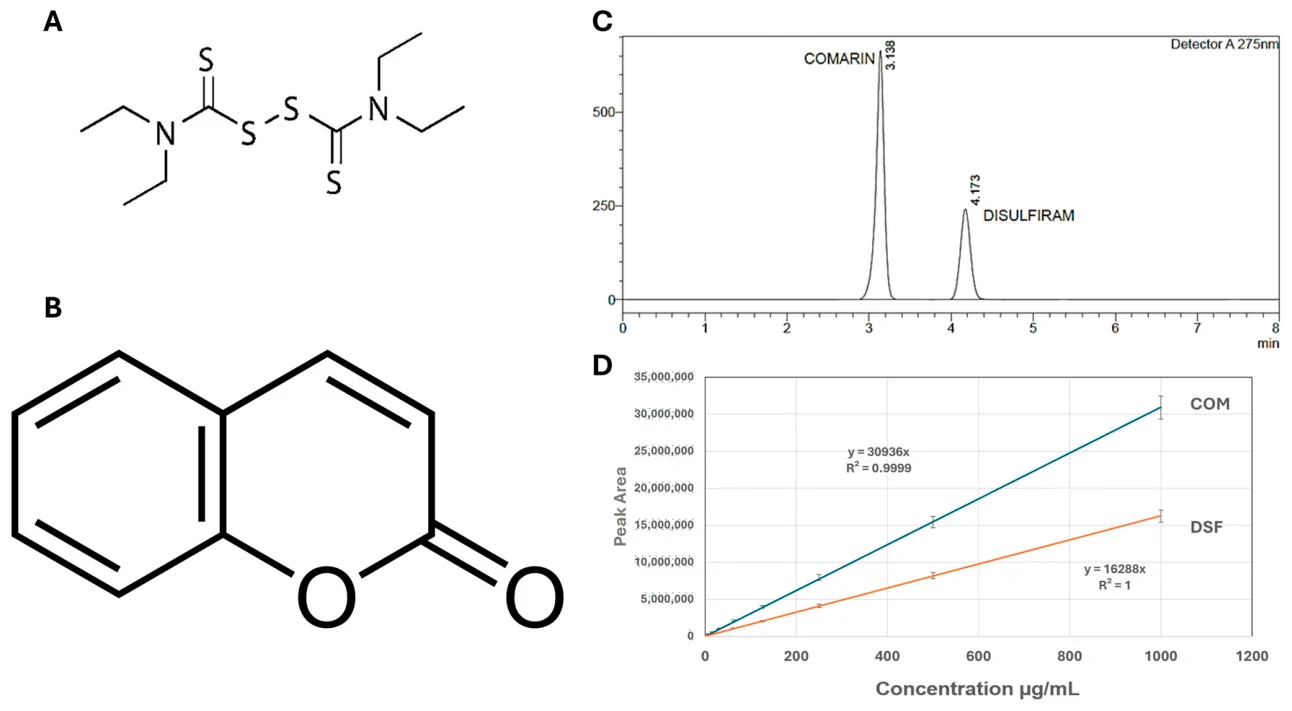
Simultaneous HPLC quantification of the active compounds. (**A**) Chemical structure of DSF. (**B**) Chemical structure of COM. (**C**) Representative HPLC chromatogram showing clear separation of COM and DSF at 275 nm, with distinct and well-resolved peaks. (**D**) Calibration curves of COM and DSF showing the relationship between peak area and analyte concentration and demonstrating good linearity of the analytical method (R^2^ > 0.99).

The HPLC chromatogram (Figure 1C) showed clear separation of coumarin and disulfiram, with distinct peaks at retention times of approximately 3.14 min and 4.17 min, respectively. No significant peak overlap was observed, indicating good chromatographic selectivity. This allowed identification and quantification of both DSF and COM within a relatively short analysis time. The calibration curves presented in Figure 1D showed a strong linear relationship between analyte concentration and peak area over the tested concentration range. The regression equations were *y* = 30,936*x* for coumarin (R^2^ = 0.9999) and *y* = 16,288*x* for disulfiram (R^2^ = 1.0000). The correlation coefficients close to 1.0 indicate excellent linearity of the method for both compounds. The higher slope observed for coumarin also indicates a greater detector response compared with disulfiram under the same chromatographic conditions.

Overall, the developed HPLC method provided good separation of coumarin and disulfiram with high linearity and a short run time. These characteristics make the method suitable for the simultaneous quantitative determination of both compounds in the developed formulations.

The experimental analysis revealed an EE% of 85.4% for DSF and 60.9% for COM. For a single 3.0 mL nanoparticle batch, these efficiencies correspond to actual encapsulated doses of 0.854 mg for DSF and 0.305 mg for COM. Consequently, the DL% was determined to be 5.69% for DSF and 2.03% for COM.

The significantly higher encapsulation efficiency and drug loading of DSF suggest that it exhibits a stronger hydrophobic affinity for the inner polymeric matrix formed during ionic complexation. Conversely, the lower incorporation parameters of COM may be attributed to its unique physicochemical characteristics, such as distinct steric hindrance or weaker molecular interactions with the hydrophilic segments of the HA-PROT complex. Nonetheless, the ionic complexation network demonstrated a robust capacity for the simultaneous loading and entrapment of both active payloads [20,25].

### 3.2. Preparation and Characterization of HA-PROT-DSF-COM NPs

**Figure 2:**
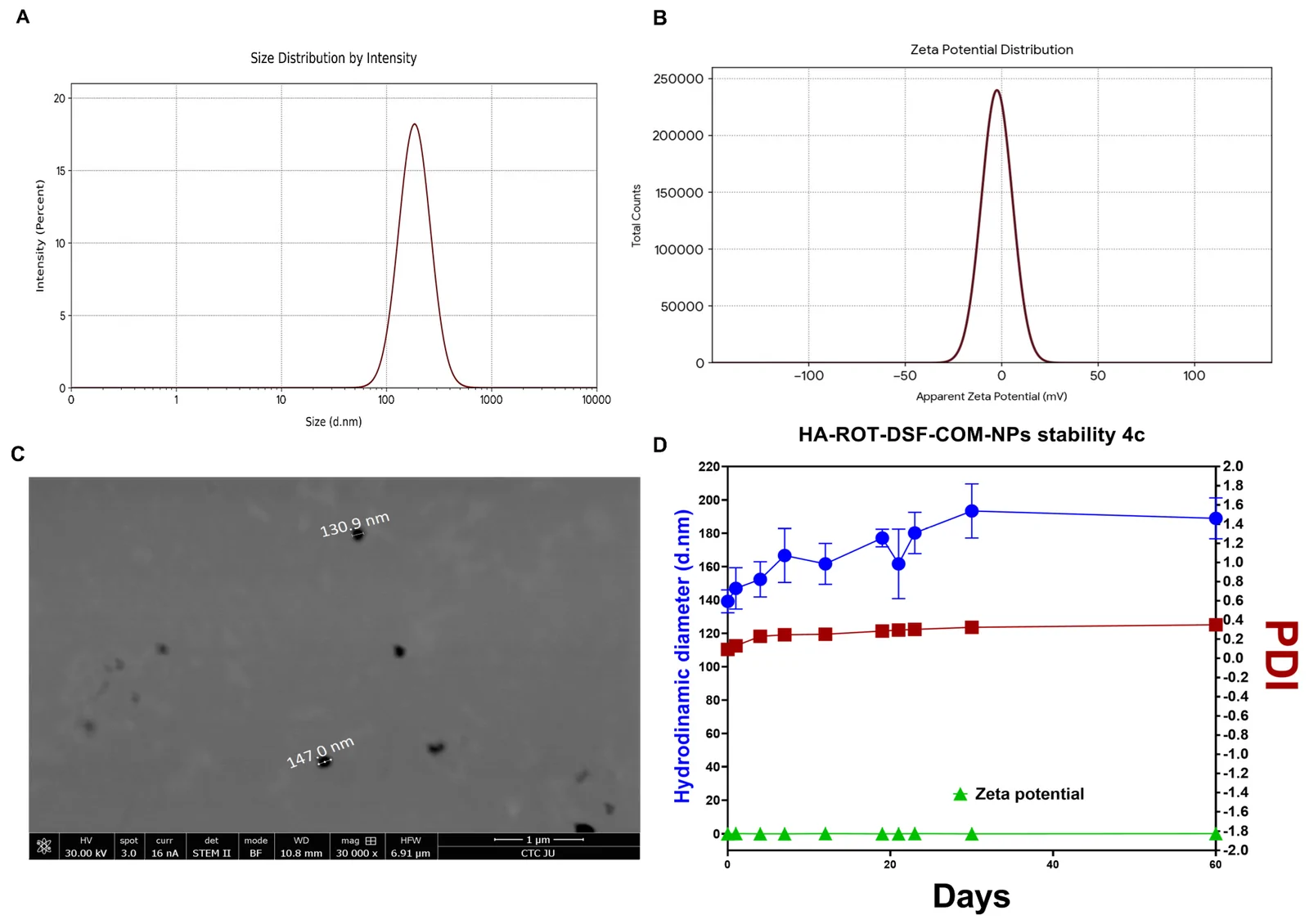
Physicochemical characterization, morphology, and storage stability of co-loaded NPs. (**A**) Hydrodynamic size distribution profile measured by dynamic light scattering (DLS) intensity percentage. (**B**) Representative apparent zeta potential distribution profile of HA-PROT-DSF-COM-NPs centered near zero. (**C**) Scanning transmission electron microscopy (STEM) micrograph confirming spherical particle morphologies and solid-state diameter attributes. (**D**) Comprehensive 60-day physical stability profile of HA-PROT-DSF-COM-NPs stored at 4 °C, tracking variations in hydrodynamic diameter (blue circles, left axis), polydispersity index (PDI, red squares, right axis), and zeta potential stability points (green triangles near the zero baseline).

The physicochemical characterization confirmed the successful preparation of HA-PROT-DSF-COM-NPs with properties suitable for topical drug delivery. The particle size distribution obtained by dynamic light scattering (DLS) showed a single, well-defined peak, with an average hydrodynamic diameter of approximately 180–200 ± 20–30 nm (Figure 2A). The relatively narrow distribution suggests good particle uniformity with limited aggregation. This particle size range may be beneficial for topical application by providing good contact with the wound surface and supporting prolonged drug retention at the application site.

The zeta potential profile showed a single, relatively narrow peak centered close to 0.02 ± 3.34 mV (Figure 2B), indicating a near-neutral surface charge. Despite the low surface charge, the formulation remained physically stable during storage. This stability may be related to steric effects provided by the hyaluronic acid and polymeric components, which can contribute to particle stabilization in addition to electrostatic interactions [26].

The morphology of the nanoparticles was further examined by transmission electron microscopy (TEM) (Figure 2C). The micrographs showed well-dispersed, nearly spherical nanoparticles, with particle diameters ranging from approximately 130 to 147 ± 10–30 nm. The smaller particle size observed by TEM compared with DLS is because TEM measures the size of dried particles, whereas DLS measures the hydrodynamic diameter of particles in dispersion [27]. The physical stability of the formulation was assessed during storage (Figure 2D). Only minor changes in particle size were observed over the study period, with no evident signs of substantial aggregation or particle growth. The PDI showed a slight increase with time but remained below 0.4, indicating that the nanoparticles maintained an acceptable size distribution throughout the storage period. The surface charge of the nanoparticles remained highly stable throughout the 2-month storage period, maintaining a narrow zeta potential of 0.02 ± 3.34 mV with no significant variations between successive measurements.

Taken together, the HA-PROT-DSF-COM-NPs showed a relatively uniform particle size, near-neutral surface charge, spherical morphology, and satisfactory physical stability. These properties support their potential use as a topical delivery system for wound-healing applications.

**Figure 3:**
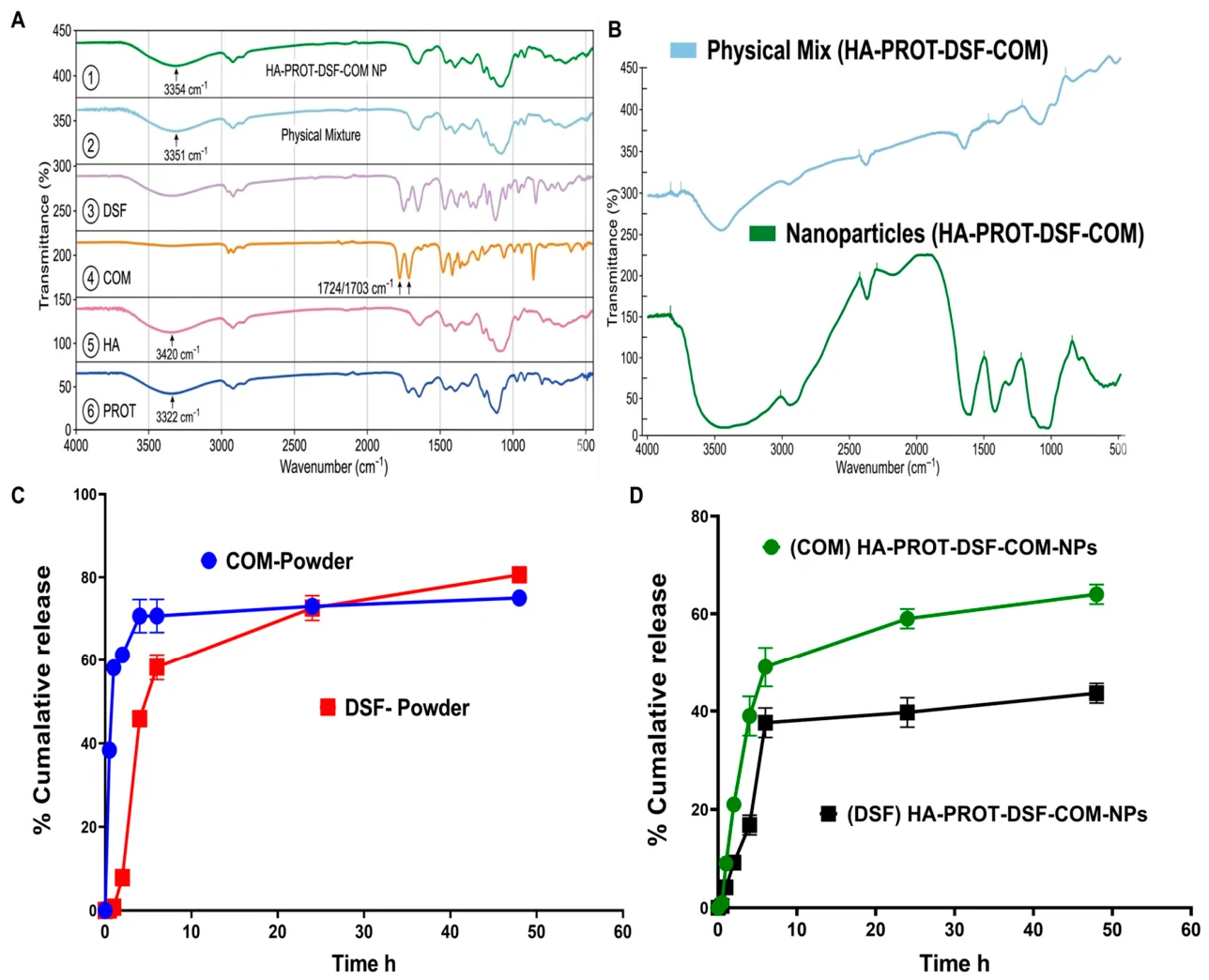
Fourier-transform infrared (FTIR) spectroscopic analysis and in vitro cumulative drug release kinetics: (**A**) comparative spectra of (1) HA-PROT-DSF-COM-NPs, (2) the physical mixture of all components, (3) DSF, (4) COM, (5) HA and (6) PROT, were recorded over the wavenumber range of 4000–500 cm^−1^. (**B**) Magnified FTIR transmittance profiles comparing the physical mixture (green line) with the synthesized HA-PROT-DSF-COM-NPs (orange line). (**C**) In vitro cumulative drug release (%) over 48 h of COM and DSF free and (**D**) Encapsulated in the HA-PROT-DSF-COM-NPs. Data are presented as mean ± SD (n = 3).

FTIR spectroscopy was performed to confirm the successful formation of the polyelectrolyte nanoparticle matrix and evaluate structural interactions between the polymeric carriers and encapsulated active drugs (Figure 3A,B). The spectrum of pure PROT exhibited a distinct characteristic band at 3322 cm^−1^, corresponding to -NH_2_ and -OH stretching vibrations, while pure HA displayed its characteristic broad polysaccharide absorption peak at 3420 cm^−1^. In the pure COM spectrum, sharp characteristic peaks emerged at 1724 cm^−1^ and 1703 cm^−1^, which are assigned to the stretching vibrations of its lactone carbonyl (C=O) group. A comparison of the raw physical mixture with the synthesized nanoparticle formulation showed noticeable changes in the characteristic absorption bands of the individual components (Figure 3B). In the HA-PROT-DSF-COM-NPs, the carbonyl stretching band of COM was markedly reduced and broadened, while the main hydroxyl/amino absorption band shifted to 3354 cm^−1^. The changes in peak position, intensity, and shape suggest interactions between the drug molecules and HA-PROT polymers during NPs formation. The attenuation of the drug peaks also supports the successful incorporation of DSF and COM into NPs rather than an unmodified physical mixture. Finally, the FTIR findings indicate good compatibility between the formulation components and provide evidence of interactions associated with nanoparticle formation. It is worth noting that sucrose contributes characteristic vibrations primarily in the O–H stretching region and the fingerprint region, particularly between 1100 and 900 cm^−1^. However, these regions overlap with vibrational bands arising from HA, protamine, DSF, and COM; therefore, individual peaks cannot be assigned exclusively to sucrose.

### 3.3. In Vitro Cumulative Drug Release

The in vitro drug release behavior of the co-loaded NPs was evaluated over 48 h to determine their ability to provide sustained release of COM and DSF (Figure 3C). The free drugs showed a relatively rapid release pattern compared with the NPs formulation. Free COM reached more than 70% cumulative release within the first 4 h, whereas free DSF reached about 60% within 6 h. This rapid release indicates that the unencapsulated drugs were readily available for dissolution in the release medium.

In contrast, the HA-PROT-DSF-COM-NPs showed a slower and more controlled release pattern for both compounds. An initial release was observed during the first few hours, followed by a more gradual increase in cumulative drug release over the remaining study period. After 48 h, around 64% of the encapsulated COM and 44% of DSF were released. The slower release from the nanoparticle formulation may be attributed to the interaction of the drugs with the polymeric matrix and the additional diffusion barrier provided by the nanoparticle structure. These findings indicate that the HA-PROT-DSF-COM-NPs can slow down the release of both drugs compared with their free forms. This may be beneficial for maintaining drug availability at the wound site over an extended period [28].

### 3.4. Preparation and Characterization of Hydrogel-HA-PROT-DSF-COM-NPs

#### 3.4.1. Swelling Behavior and Spreadability

**Figure 4:**
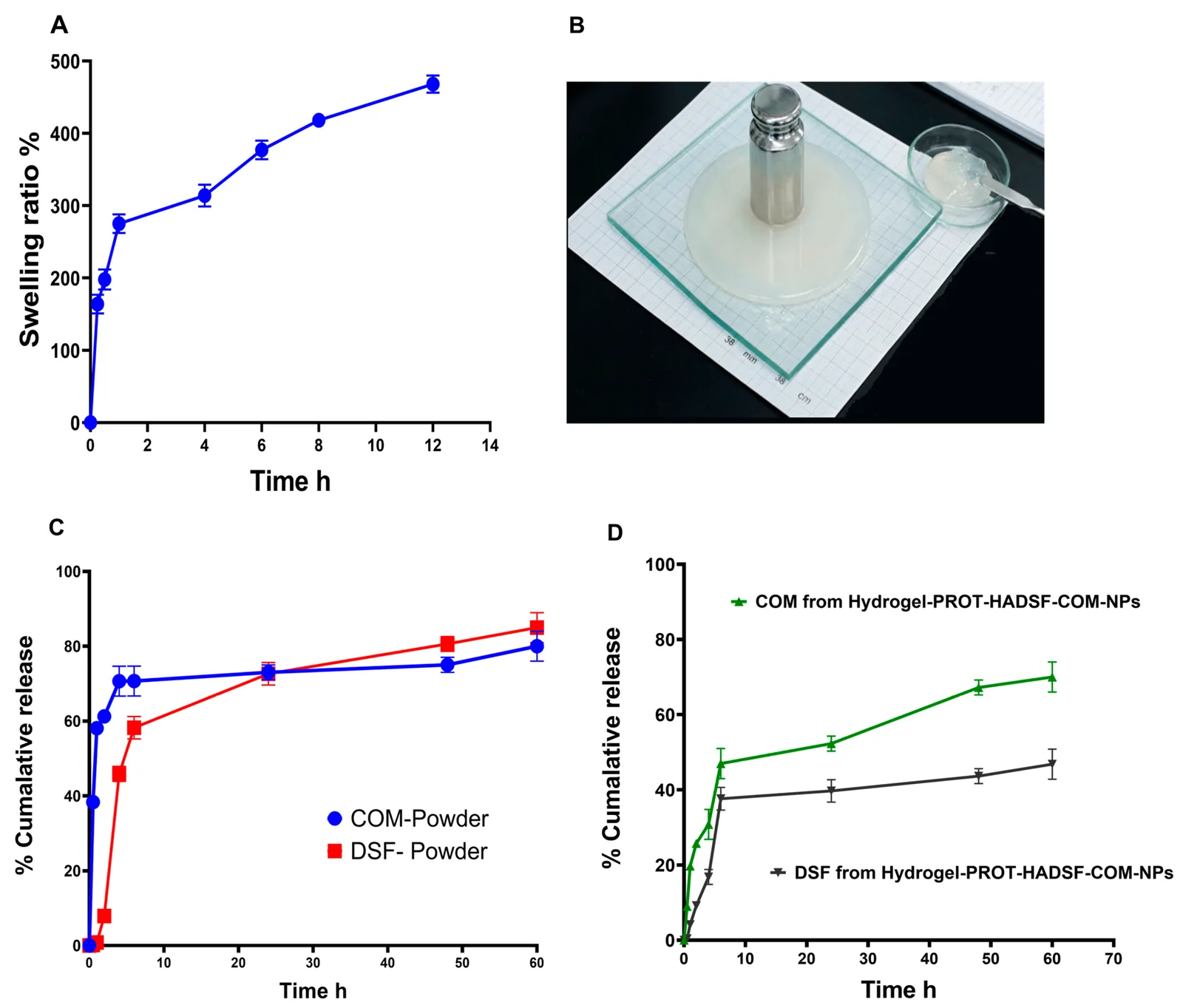
Swelling behavior, in vitro drug release kinetics, and macroscopic texture of the hydrogel. (**A**) Swelling ratio percentage (%) of the hydrogel as a function of time. (**B**) Photographic representation demonstrating the macroscopic appearance, transparency, and mechanical structural integrity/spreadability of the formulated hydrogel under a standard physical weight. (**C**) Cumulative in vitro release profiles (%) over 60 h evaluating COM and DSF in their free powder forms. (**D**) Cumulative in vitro release profiles (%) over 60 h evaluating COM and DSF from the Hydrogel-HA-PROT-DSF-COM-NPs.

The swelling behavior of the optimized CMC–PG hydrogel containing HA-PROT-DSF-COM-NPs was evaluated over 12 h. As presented in Figure 4A, the hydrogel demonstrated a biphasic swelling profile. During the first hour, a rapid increase in water uptake was observed, with the swelling ratio reaching 275%. This initial expansion was attributed to the hydration of hydrophilic groups within the CMC network, which facilitated water penetration into the polymer NPs. Subsequently, the swelling rate gradually slowed, reaching a maximum swelling ratio of 468% after 12 h. The hydrogel maintained its structural integrity throughout the experiment, with no visible erosion or disintegration. This stability can be related to the presence of PG, which enhanced flexibility while preserving the interactions between CMC chains. The hydrogen bonding and polymer chain entanglement contributed to the mechanical stability and allowed sustained hydration without compromising the hydrogel structure [29]. The spreadability of Hydrogel-HA-PROT-DSF-COM-NPs was assessed to evaluate its suitability for topical application (Figure 4C). The formulation demonstrated smooth and uniform spreading, with a diameter of 18 ± 1.2 mm under the weight of the upper glass plate and increasing to 38 ± 1.5 mm after applying a 50 g load. This spread diameter is within the desirable range reported for topical hydrogels (30–50 mm), indicating that the formulation can be easily applied while maintaining adequate retention at the site of administration. The CMC provided suitable viscosity to prevent excessive spreading, whereas PG improved the flexibility of the polymer by modulating interactions between CMC chains. These properties allowed the hydrogel to spread evenly with minimal force, supporting comfortable application on wound surfaces and promoting uniform distribution of the encapsulated therapeutic agents [30]. The degradation profile and integrity of the nanoparticle-embedded 2% *w*/*w* CMC hydrogel can be structurally concluded from its swelling kinetics and long-term physical stability profile. The highly controlled swelling equilibrium highlights a robustly cross-linked polymeric network that resists rapid hydrolytic dissociation. This behavior corresponds to a highly desirable, controlled *bulk erosion* degradation profile, where the structural disassembly of the carboxymethyl cellulose framework occurs gradually rather than through sudden bulk collapse. This conclusion is strongly corroborated by the 60-day storage stability data; the retention of the matrix network and the stable near-neutral zeta potential over 2 months confirm that the secondary CMC matrix prevents premature enzymatic or hydrolytic breakdown of the embedded HA-PROT nanoparticles. This synchronized degradation profile ensures that the hydrogel remains an intact localized depot capable of supporting sustained, prolonged drug release at the application site.

#### 3.4.2. In Vitro Release Kinetics

The in vitro release profiles demonstrate that encapsulating drugs in the Hydrogel-HA-PROT-DSF-COM-NPs significantly alters their mass transport kinetics compared to their unencapsulated, free powder forms (Figure 4C,D). As shown in Figure 4C, the free COM and DSF powders exhibited rapid, uncontrolled dissolution with an immediate burst phase, reaching ~70% (within 4 h) and ~80% (by 60 h) cumulative release, respectively, due to unrestricted exposure to the aqueous medium. In contrast, encapsulation within the hydrogel network generated distinct, drug-specific release profiles (Figure 4D) rather than uniform behavior for both payloads. During the initial 6 h, surface-associated drug molecules rapidly diffused into the medium, though the formulation successfully moderated this burst by restricting release to ~47% for COM and ~38% for DSF. Following this initial phase, the subsequent kinetics deviated significantly. COM demonstrated a true, prolonged sustained release pattern, steadily diffusing upward to liberate ~70% of its payload by 60 h, whereas DSF failed to exhibit sustained release over time, abruptly plateauing after 6 h and climbing marginally to a maximum of only ~47% by the end of the study [31]. Following this initial desorption phase, the transport kinetics of the two co-loaded payloads from the hydrogel, which incorporates both the embedded nanoparticles and the unencapsulated powder fractions, diverged sharply, demonstrating that they do not share a uniform matrix-release behavior over the 48 h window. This sustained behavior is driven by a dual-barrier mechanism where COM must diffuse out of the nanoparticles and subsequently migrate through the crosslinked hydrogel network; when fitted to the classical Higuchi kinetics model, this profile demonstrated a linear correlation coefficient (R^2^ = 0.984), confirming a highly stable, matrix-controlled Fickian diffusion mechanism through the pores of the hydrogel loaded with DSF and COM. Conversely, the DSF component within the hydrogel network failed to adhere to a continuous sustained-release profile, abruptly flatlining after 6 h and shifting marginally from ~38% to a terminal maximum of only ~43% at 48 h. Consequently, the later phase of DSF release deviates significantly from the Higuchi power law, yielding a poor overall kinetic fit (R^2^ = 0.712), which indicates that while the unencapsulated powder fraction near the gel surface contributes to the initial burst, the remaining fraction of loaded DSF is heavily restricted, staying tightly sequestered within the hydrophobic core of the nanoparticles or bound by powerful intermolecular forces along the polymer backbones, restricting its outward diffusion under the tested sink parameters.

### 3.5. Biological Characterization

#### 3.5.1. In Vitro Cytotoxicity and Scratch Assay

**Figure 5:**
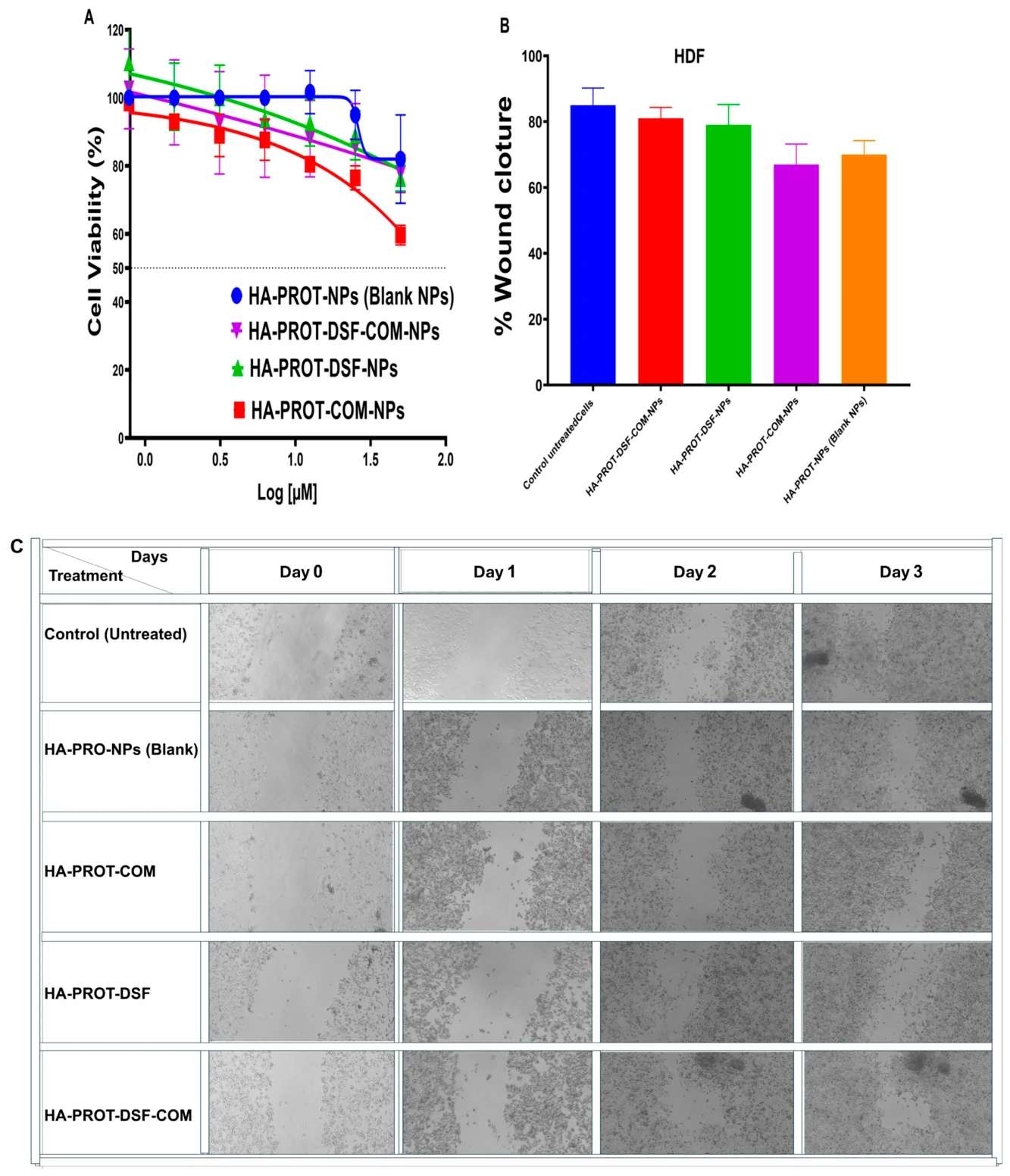
In vitro cytotoxicity and quantitative wound closure analysis on HDFs: (**A**) Cell viability percentage (%) of HDFs after treatment. (**B**) Quantitative percentage (%) of wound closure in HDFs across different experimental groups for the HA-PROT-DSF-COM-NPs compared to untreated control cells and single-drug counterparts (*p* < 0.05). (**C**) In vitro scratch wound healing assay evaluating cellular migration. Representative optical micrographs capturing the progressive closure of an artificial scratch wound across different treatment groups over a 3-day timeline (Day 0, Day 1, Day 2, and Day 3). The rows compare cellular migration rates under various experimental conditions: untreated control, HA-PROT-NPs (Blank), HA-PROT-COM-NPs and HA-PROT-DSF-NPs, and HA-PROT-DSF-COM-NPs.

The cell viability assay was performed for all formulations using the human dermal fibroblast (HDF) cell line in a concentration range of 0.78–50 µM (Figure 5A). Blank HA-PROT-NPs, HA-PROT-DSF-NPs, HA-PROT-COM-NPs and HA-PROT-DSF-COM-NPs showed no cytotoxicity even at the highest concentrations tested. The high cell viability which was above 80% confirms the formulations’ safety.

To determine the therapeutic efficacy of the formulations on tissue repair, an in vitro scratch wound assay was performed over a 3-day developmental timeline (Figure 5C). Visual inspection of the optical micrographs revealed a progressive, time-dependent narrowing of the initial scratch gap across all treated groups from day 0 to day 3 [32,33]. While the untreated control and blank NP groups exhibited incomplete closure, groups treated with HA-PROT-COM-NPs and HA-PROT-DSF-NPs also showed slower cellular migration. The HA-PROT-DSF-COM-NPs achieved relatively comparable wound closure by Day 3. Quantitative analysis of the wound closure percentage confirmed these visual findings (Figure 5B,C), establishing that the HA-PROT-DSF-COM-NPs enhanced HDF migration and proliferation.

In this specific 2D design, the untreated control cells show rapid horizontal migration into the center of the scratch on Day 3. While the control cells crawled quickly across the gap, our target formulation (HA-PROT-DSF-COM-NPs) stimulated a different cellular behavior characterized by massive cell proliferation, high cell density, and robust, thick monolayer formation along the margins. The treated groups contain nanoparticle-based formulations, and their effects may depend on the interaction and release of the incorporated components over time. Therefore, the cellular response may not necessarily follow the same kinetics as the untreated control. The apparent difference on Day 3 should consequently be interpreted together with the quantitative wound-closure measurements rather than from the representative images alone. We have clarified this point in the revised manuscript [34,35].

#### 3.5.2. In Vivo Wound Healing Assay

**Figure 6:**
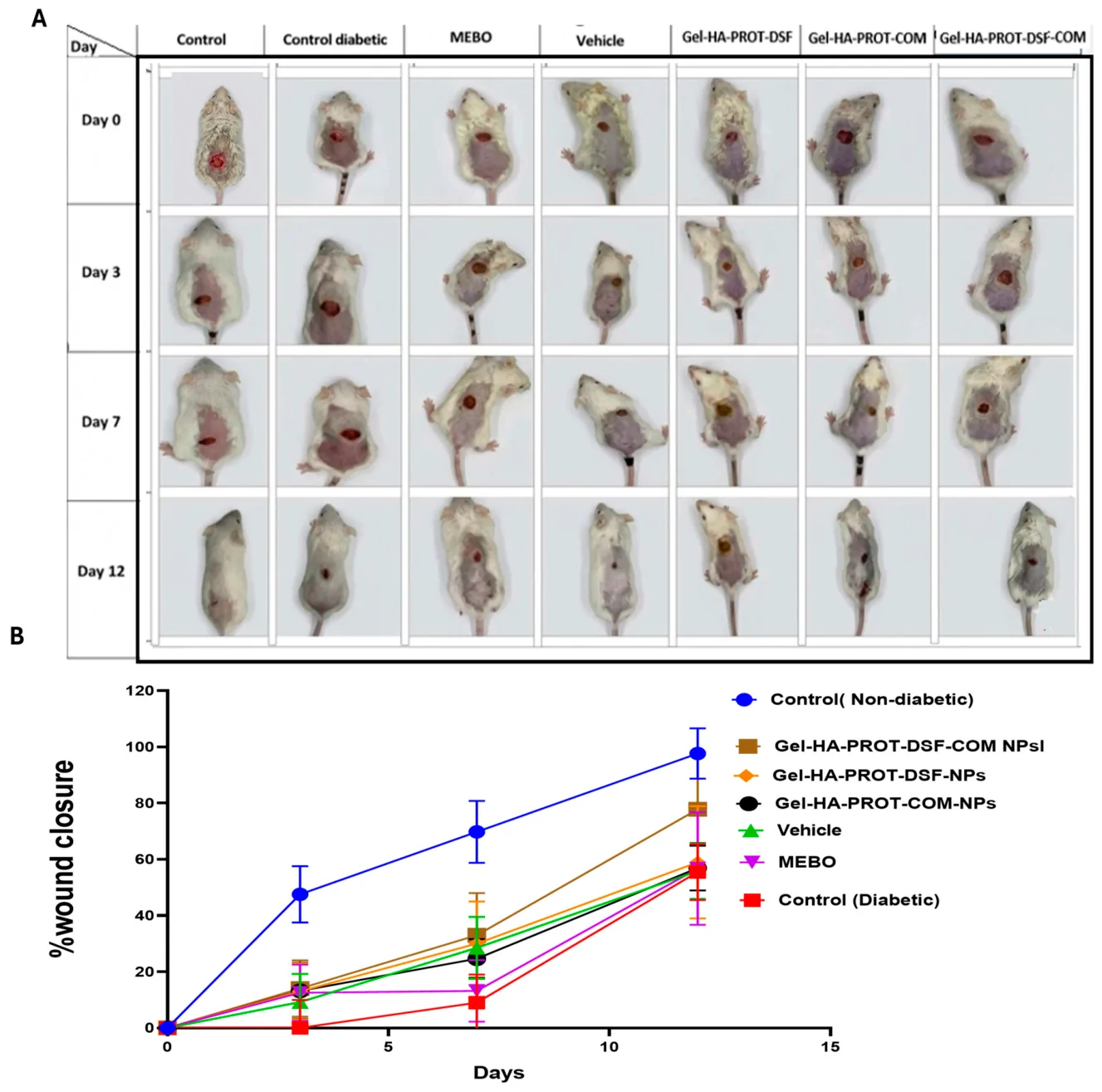
(**A**) In vivo wound healing assessment in diabetic mice. Representative dorsal photographs of full-thickness excisional wounds at Days 0, 3, 7, and 12 post-treatment across seven experimental groups: Control, MEBO, Vehicle, Gel-HA-PROT-DSF-NPs, Gel-HA-PROT-COM-NPs and Gel-HA-PROT-DSF-COM-NPs. (**B**) Progressive wound closure %.

To assess the therapeutic efficacy of the formulations in wound healing, wounded diabetic mouse models were developed and tracked longitudinally over 12 days (Figure 6A,B). The healthy non-diabetic group demonstrated rapid and linear healing, achieving 48% wound closure by day 3 and near-total closure of about 97% by Day 12. Conversely, the diabetic group exhibited severe pathological impairment, presenting 0% wound closure at day 3. Among the experimental treatments administered to the diabetic models, the DSF formulation was the top therapeutic candidate, accelerating healing to 21% on day 3 and yielding the highest diabetic wound contraction of 82% by day 12. Importantly, combining DSF with COM produces enhanced wound repair to reach 78% closure. Meanwhile, commercial MEBO yields quantitative closure rates of 56.7%, nearly identical to the vehicle hydrogel.

#### 3.5.3. Histopathological Analysis of Diabetic Wound Healing

**Figure 7:**
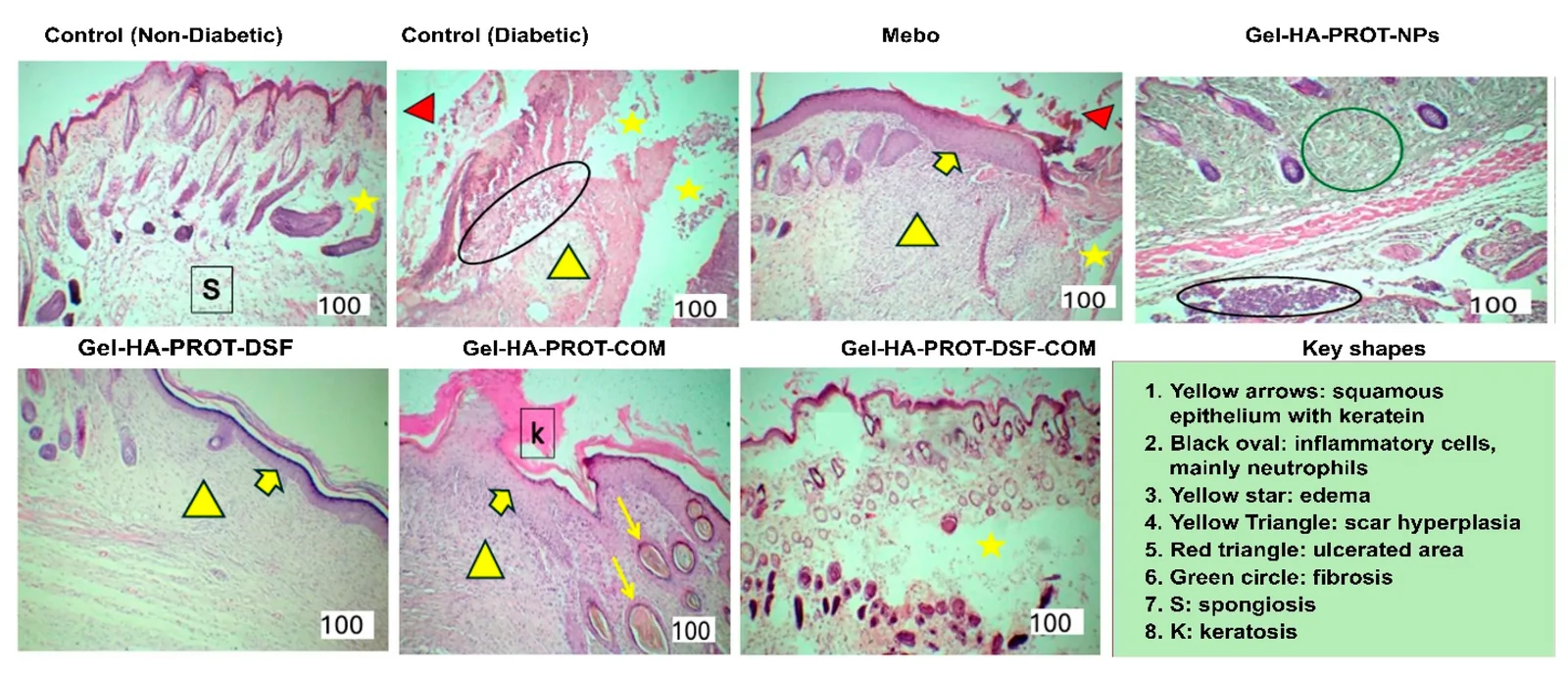
Representative H&E-stained skin sections of wounds from the experimental groups (100×). The non-diabetic control showed normal skin architecture, whereas the diabetic control exhibited ulceration, edema, inflammatory cell infiltration, and scar hyperplasia. Mebo and Gel-HA-PROT-DSF improved re-epithelialization and reduced inflammation. Gel-HA-PROT-COM promoted epidermal regeneration and hair follicle formation. Gel-HA-PROT-DSF-COM showed the greatest wound healing, with nearly complete re-epithelialization and restoration of normal skin architecture. Histological markers: yellow arrows, keratinized epithelium; black ovals, inflammatory cells; yellow stars, edema; yellow triangles, scar hyperplasia; red arrowheads, ulceration; green circles, fibrosis; S, spongiosis; K, keratosis. Scale bar = 100 μm.

Histopathology of skin sections was performed to evaluate wound-healing progression in diabetic mice treated with hydrogel formulations. As shown in Figure 7, the diabetic control mice reveal the classic triad of chronic diabetic wound pathology which includes persistent ulceration (red triangle), neutrophil-predominant inflammation (black oval), and edema (yellow star). In a normal healthy acute wound healing process, neutrophils rapidly clear within a few days, and their removal directly facilitates macrophage activity and tissue repair [6]. In the diabetic mice, the dense neutrophilic infiltrate indicates an inflammatory phase that has failed to resolve [36]. While the scar hyperplasia (yellow triangle) reveals dysregulated remodeling of myofibroblasts. As a hallmark of diabetes-impaired wound healing, the absence of re-epithelialization confirms that keratinocyte migration from wound edges is arrested [37].

The hydrogel that contains blank NPs, Gel-HA-PROT-NPs, is associated with a relatively well-organized tissue structure, with evidence of tissue regeneration and reduced inflammatory changes. The epidermal and dermal regions appear more organized, while the presence of developing hair follicles and skin appendages suggests ongoing tissue repair. The circled area shows relatively loose and less cellular tissue, which may indicate mild residual edema of the dermis. The black-circled region contains a more cellular area, which may represent residual inflammatory cells or developing granulation tissue. The histological appearance suggests active wound repair with relatively good tissue organization and limited inflammatory response, supporting the potential beneficial effect of Gel-HA-PROT-NPs in promoting diabetic wound healing [38].

Mebo, the moist exposed burn ointment, shows partial re-epithelialization (yellow arrow), consistent with its established role in promoting keratinocyte migration and granulation tissue formation. However, the persistent ulceration (red triangle), edema, and scar hyperplasia indicate that while MEBO accelerates epithelialization, it does not fully resolve the underlying chronic inflammation [39].

Gel-HA-PROT-DSF demonstrates the most mature stratified squamous epithelium (yellow arrow) among the single-agent groups, with clear keratinization suggesting successful terminal differentiation of keratinocytes. This implies that the DSF component potently activates the proliferative phase, specifically keratinocyte migration and stratification. The scar hyperplasia (yellow triangle) in the underlying dermis reveals that a disconnected epidermal coverage is achieved without proper remodeling of the granulation tissue. Pathologically, this is significant; it suggests DSF accelerates epithelial closure but does not adequately modulate myofibroblast activity or ECM turnover, leaving the wound mechanically weak and functionally compromised despite surface closure [18,40].

The treatment that contained COM, Gel-HA-PROT-COM-NPs, is linked with re-epithelialization and ongoing tissue remodeling. The epidermis shows squamous epithelium with keratin formation, as indicated by the yellow arrows. Keratosis is also observed in the area marked K, while spongiosis (S) indicates intercellular edema within the epidermal layer. The dermis shows edema, highlighted by the yellow star, together with scar hyperplasia indicated by the yellow triangle. Inflammatory cell infiltration, mainly neutrophils, is present in the black-circled area, suggesting that some inflammatory activity persists. The section also shows an ulcerated area, marked by the red triangle, and areas of fibrosis, represented by the green circle. As shown in Figure 7, the histological section demonstrates partial tissue repair with re-epithelialization and epidermal keratinization, accompanied by persistent inflammation, edema, fibrosis, and scar formation [41,42].

The treatment that combined DSF and COM, Gel-HA-PROT-DSF-COM, stands out as the only group approaching the non-diabetic control mouse model. The epidermis appears thin, stratified, and appropriately keratinized without the excessive hyperkeratosis seen in COM alone. Multiple hair follicles are organized in a near-normal pattern, indicating restoration of adnexal structures, a standard of true regenerative healing rather than mere wound closure. The residual edema (yellow star) suggests low-grade vascular permeability or lingering inflammation, but the absence of ulceration, neutrophilic infiltrates, dense fibrosis, or scar hyperplasia is remarkable. As detailed in Table 1, semi-quantitative evaluation based on the Abramov and modified Greenhalgh scoring guidelines tracked a clear hierarchical improvement in wound tissue architecture across the cohorts. The scores progressed systematically from the severely compromised state of the untreated Diabetic Control group (1/5), through intermediate repair stages observed in the Mebo and single-agent nanoparticle cohorts (3/5 to 3.5/5), culminating in the advanced structural recovery demonstrated by both the HA-PROT-DSF-NPs and HA-PROT-DSF-COM-NPs treatment groups (4/5).

DSF provides the epithelialization drive that COM alone lacks, while COM contributes regenerative signaling, such as angiogenesis and adnexal induction, that DSF alone cannot deliver [19,40,42]. The Gel-HA-PROT-NPs serve as a hydrated scaffold enabling sustained release. The combination normalizes both the epidermal and dermal compartments, achieving what appears to be the closest approximation to acute wound resolution in a diabetic microenvironment [43,44]. True healing requires coordination of both compartments. The combination corrects monotherapy limitations. The COM has a tendency toward hyperkeratosis, normalized when combined with DSF, while DSF’s scarring tendency is ameliorated by COM. This suggests the two payloads modulate distinct but complementary pathways, likely keratinocyte differentiation/migration DSF and angiogenesis/dermal regeneration COM [43]. Even in the best-performing DSF-COM group, edema persists. This suggests that while cellular inflammation resolves, vascular permeability or lymphatic dysfunction is the last component of the diabetic wound microenvironment to normalize, a clinically relevant observation for predicting recurrence risk [42].

## 4. Conclusions

Histopathological evaluation demonstrated that the developed nanoparticle-embedded hydrogels supported positive tissue changes during diabetic wound repair. Diabetic control wounds exhibited severe tissue damage, incomplete epithelialization, necrotic changes, and poor dermal organization, confirming impaired healing under diabetic conditions. In contrast, groups treated with the developed nano-formulations showed tissue regeneration characteristics, including advanced re-epithelialization, organized collagen arrangement, lower inflammatory signs, and visible dermal remodeling. Specifically, Gel-HA-PROT-DSF-NPs supported regenerative patterns characterized by continuous epithelial coverage and minimal inflammation. Similarly, the dual-cargo Gel-HA-PROT-DSF-COM-NPs formulation was associated with tissue restoration, showing regenerated epidermal structures, and organized dermal layers.

## 5. Limitations and Future Perspectives

While the current study successfully validates the synthesis, dual-cargo release behavior, and in vivo therapeutic efficacy of the Gel-HA-PROT-DSF-COM-NPs, certain macro-structural limitations remain to be addressed. Due to regional equipment and core infrastructure boundaries, explicit rheological profiling, mechanical shear testing, and long-term enzymatic degradation kinetics of the hydrogel carrier were not executed in this foundational stage. Furthermore, while standard histopathological analysis conclusively demonstrated advanced tissue remodelling and adnexal development, isolated quantitative tracking of extracellular matrix density via specialized collagen stains or biochemical assays was restricted. Future investigations during the industrial scale-up and optimization phases will focus on resolving these mechanical characteristics and evaluating extended pharmaceutical shelf-life stability to fully prepare this platform for clinical translation.

## Figures and Tables

**Table 1:** Semi-quantitative histopathological evaluation and global healing scores of excisional wound tissues across the different experimental cohorts on Day 12, adapted from the Abramov grading criteria (n = 6).

Group (×100)	Histological Features	Healing Score	Interpretation
Control (Diabetic)	Severe tissue disruption, incomplete epithelial coverage, necrotic/degenerated areas, poor dermal organization	1/5	Very poor healing
HA-PROT-COM-NPs	Fair dermal organization, limited epithelial thickening, moderate remodeling	3/5	Moderate healing
Mebo Ointment	Thick re-epithelialization with persistent inflammatory infiltrate and dermal cellularity	3/5	Intermediate healing
HA-PROT-NPs (Blank NPs)	Hyperkeratosis and thickened epidermis, moderate remodeling, residual irregularity	3.5/5	Good but reactive healing
HA-PROT-DSF-NPs	Thin but continuous epithelium, relatively mature collagen alignment, low inflammation	4/5	Good remodeling/healing
HA-PROT-DSF-COM-NPs	Good epidermal regeneration with preserved adnexal structures; moderate dermal remodeling	4/5	Advanced healing
Control (Non-Diabetic)	Excellent epithelial regeneration, abundant hair follicles, organized dermis	5/5	Near-complete healing

## Data Availability

The raw and processed data supporting the findings of this study are available from the corresponding author upon reasonable request.
